# Cancer survivorship in urban people living with cancer following primary treatment: A secondary analysis of qualitative interview data

**DOI:** 10.1007/s00520-024-08464-9

**Published:** 2024-04-02

**Authors:** Saood Mahmood, Florence Graham, Samuel Cooke, Ros Kane, David Nelson

**Affiliations:** 1 Lincoln Medical School, Universities of Nottingham and Lincoln, Lincoln, UK; 2 College of Health and Science, Lincoln International Institute for Rural Health, University of Lincoln, Lincoln, LN6 7TS UK; 3 School of Health and Social Care, University of Lincoln, Lincoln, UK; 4 Macmillan Cancer Support, London, UK

**Keywords:** Cancer survivorship, Urban health, Interview data, Qualitative study, United Kingdom

## Abstract

**Purpose:**

Urban cancer survivors have been shown to have better opportunities for recovery of health and wellbeing than their rural counterparts. Whilst there is a considerable body of evidence that explores urban people with cancers’ experiences and outcomes, there is a dearth of research that explicitly explores ‘urban cancer survivorship’ in its own right. This study aimed to explore cancer survivorship in urban people living with cancer who have completed primary treatment.

**Methods:**

Secondary analysis of in-depth interview data (*n* = 18) with adults living with cancer who resided in urban parts of the UK. Data were drawn from a broader study on self-management of people living with cancer. An adapted version of Foster and Fenlon’s recovery of health and wellbeing in cancer survivorship framework was used to inform the analysis of the data.

**Results:**

Recovery of health and wellbeing was impacted by a variety of contributory factors, which had a largely positive impact. Access to amenities, social support, travel, and healthcare factors were opportunities for urban cancer survivors, whilst pollution, traffic and a lack of green spaces acted as challenges for health management.

**Conclusion:**

This study demonstrated how urban residency acted as both a barrier and a facilitator to recovery of health and wellbeing in urban cancer survivors following the completion of primary treatment. Area of residence should be taken into account by health providers and policymakers supporting cancer survivorship and the views of those with lived experiences should be included in informing future practice.

## Introduction

Globally, cancer incidence has been increasing exponentially [1]. In the UK, rates of cancer have been rising each year with over 375,000 diagnoses between 2015 and 2018 [2]. In addition, the number of UK people surviving cancer has doubled over the last 40 years [2], and with 40% of cancer survivors expressing abandonment post active treatment [3], the survivorship experience is becoming an increasingly integral part of cancer care. Whilst there is no universal definition of ‘cancer survivor’ or ‘cancer survivorship’ and definitions and terminology can vary by geographic setting [4–7]. In this study, we use the term survivorship to refer to the post-treatment experiences of people who had completed primary treatment within the last five years and were not in receipt of active treatment. The terms ‘living with cancer’ and ‘living with and beyond cancer’ are also becoming increasingly used, particularly in the UK [8, 9], in that people live with the consequences of a cancer diagnosis for the rest of their life regardless of their treatment trajectory and outcomes.

In the UK, urban areas are defined as settlements with populations over 10,000, in which ~ 80% of the total population reside [10, 11]. Due to their geography and population density, urban residents typically have easier access to specialist healthcare, support groups, public transport and other amenities compared to their rural counterparts. Whilst urban residents are more likely to experience longer term survival and generally better outcomes from cancer when compared to those living in rural areas, urban cancer survivors can experience unique challenges relating to the intersectionality between cancer recovery and geography, for example, air and noise pollution and poorer mental health, as well as having access to fewer green spaces than those living in rural areas [12–15].

Recovery of health and wellbeing following primary cancer treatment is a multi-faceted process, which is often unique to the individual. A range and mixture of physical, psychological, environmental, and social issues may arise during recovery. These include fatigue, pain, impaired sexual functioning, fear of recurrence, post-traumatic stress disorder, depression, impaired cognition, loss of confidence, stress, financial difficulties, and employment concerns [16–20]. Survivors emphasise the importance of support groups and self-management along with family and friends in aiding them through their recovery [16]. Whilst there is a considerable body of evidence that explores urban people with cancers’ experiences and outcomes, there is a dearth of research that explicitly explores ‘urban cancer survivorship’ in its own right.

The aim of this study was to gain an in-depth understanding of cancer survivorship in solely urban people living with cancer who had completed primary treatment with a view to better understanding the opportunities and challenges that urban people living with cancer can face during recovery.

## Methods

### Design and procedure

This is a secondary analysis study of a cross-sectional mixed-methods study that collected quantitative and qualitative data on the experiences of cancer survivors from rural and urban settings [21–24]. The original data consisted of in-depth qualitative interviews (*N* = 34) with both urban and rural cancer survivors [21]. This secondary analysis draws specifically on interview transcripts from urban participants (*N* = 18), with a focus on how their urban residence may have impacted their recovery of health and wellbeing following primary cancer treatment. Urban/rural residence was categorised using postcodes, measured using the Office for National Statistics RUC2011 classification [11]. Deprivation was measured using postcode and aligning with the Index of Multiple Deprivation Decile [25]. This study was reported based on the Consolidated Criteria for Reporting Qualitative Research (COREQ) [26].

### Sample

Participants were recruited via Cancer Centre staff at two NHS acute trusts in the East Midlands region of England. Inclusion criteria for participants were (1) aged 18 and above (2) cancer diagnosis recorded on participating NHS trusts patient database, (3) good command of the English language (4) completed primary cancer treatment in the 5 years prior to interview, and (5) able to give informed consent, and additional criterium for selection into this secondary analysis: (6) currently residing in an urban area.

The participants for this secondary qualitative analysis were originally recruited from a self-reported postal questionnaire with an option to give consent to taking part in a further qualitative interview and to provide full informed consent for using that interview transcript in future research [21]. Interviews were conducted either face-to-face or via telephone and were conducted in the participants home or on university premises, depending on the participants preference. The interviews were undertaken by an experienced qualitative researcher who had expertise in interviewing people affected by cancer (DN). A semi-structured approach was used to provide flexibility in exploring participants’ experiences of self-management and their health and wellbeing following primary treatment. Participants were encouraged to elaborate on experiences unique to their own situation in relation to what they considered opportunities or challenges to their recovery following cancer treatment. This provided a more relaxed, natural experience for the participants and allowed elaboration of the areas important to the them [27]. Furthermore, a semi-structured approach allowed for participant self-reflection, aiding them in how they viewed their journey as a whole and may have brought up new avenues for them in facilitating their future recovery.

### Framework

**Fig. 1:**
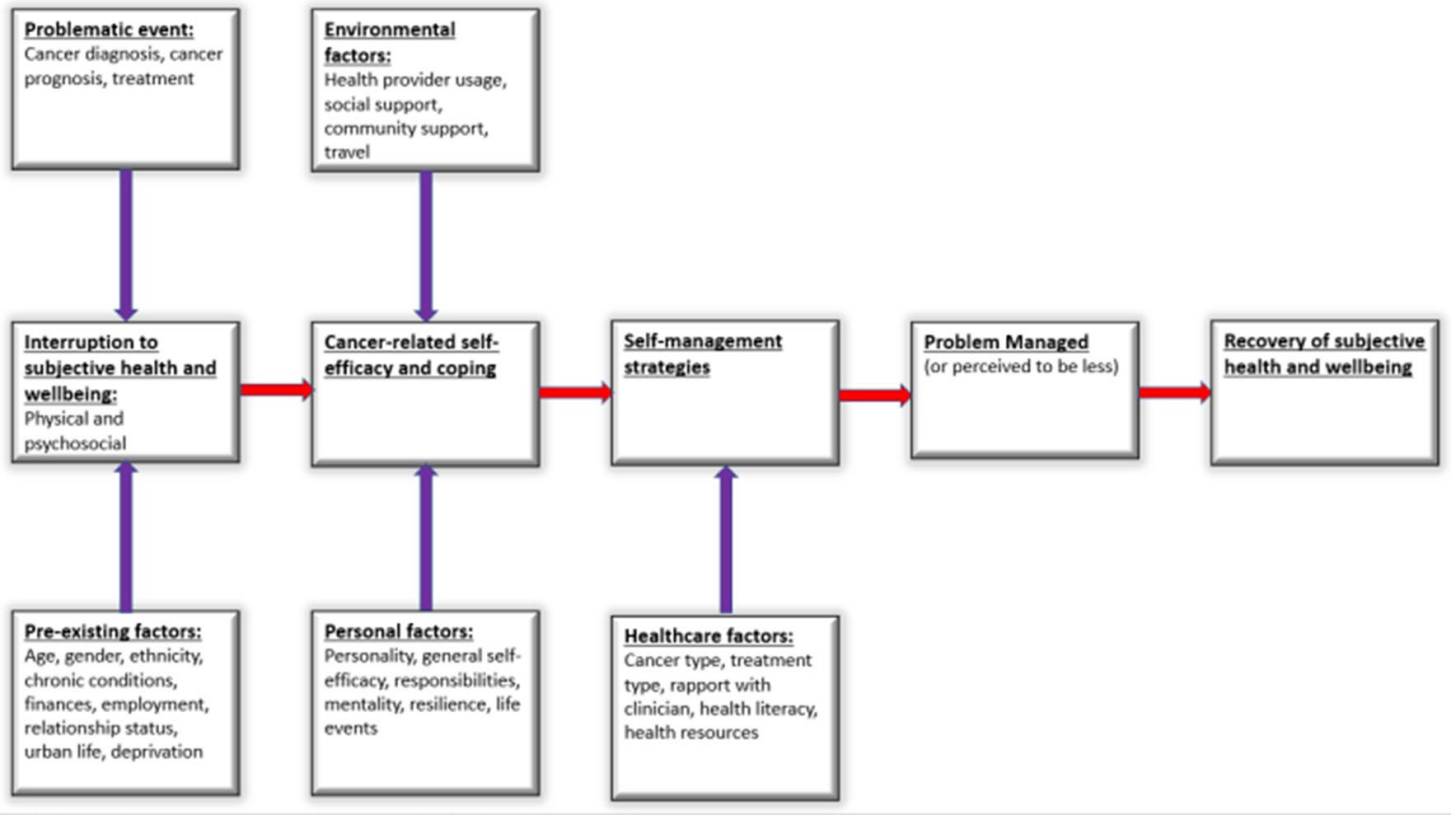
An adapted framework from Foster and Fenlon [10] showing recovery of subjective health and wellbeing in urban cancer survivors following primary cancer treatment

A framework was developed, adapted from Foster and Fenlon’s recovery of health and wellbeing in cancer survivorship framework [16], this adapted version is shown below in Fig. 1. Domains added included a more comprehensive range of pre-existing factors: ethnicity, chronic conditions, finances, relationship status and deprivation; as well as responsibilities, life events and mentality [28–31]. Healthcare factors were also added, to ensure their pivotal role was used to better understand their influence on participants and the role they play in recovery [16, 32].

### Analysis

A thematic analysis approach [33] was used to analyse the data and was chosen as the interview transcripts were very in-depth and rich in data. Use of thematic analysis allowed for a deeper understanding of the lived experience of urban cancer survivors. The initial phase of analysis involved familiarisation [34] in which data was read and re-read multiple times to ensure compatibility with the framework, as well as searching for new emerging themes within transcripts.

Coding was carried out within NVivo 12, allowing for a systematic approach to be used. Nodes were created using the main headings from the adapted theoretical framework, and child nodes subsequently comprised of the various sub-factors encompassing the main factor. A hybrid inductive/deductive reasoning approach was used [35], as transcripts were analysed chronologically into deductive codes, with data sometimes fitting into multiple nodes. The development of new emerging themes that arose were then added to the framework being used and then retrospectively searched for in transcripts that had already been analysed.

## Results

### Demographics

Eighteen participants were selected for secondary analysis based on urban residence, of which ten were male and eight were female. All participants were from the White British ethnic background and aged between 44 and 76. Participant demographic and cancer type data are provided in Table 1.

**Table 1:** Participant characteristics

Demographic category	*n* (%)
*Gender*	
Male	10 (55.6)
Female	8 (44.4)
*Age category*	
35–44	1 (5.6)
45–54	4 (22.2)
55–64	2 (11.1)
65–74	9 (50.0)
75 +	2 (11.1)
*Living arrangement*	
Live alone	7 (38.9)
Live with partner/spouse/family/friends	11 (61.1)
*Marital status*	
Divorced/single/separated/widowed	9 (50.0)
Married/civil partnership	9 (50.0)
*Employment status*	
Employed	6 (33.3)
Unemployed/retired/other	12 (66.7)
*Annual household income*	
£0–14,999	3 (16.7)
£15,000–24,999	6 (33.3)
£25,000–49,999	8 (44.4)
£50,000–74,999	0 (0.0)
Above £75,000	1 (5.6)
*Educational attainment*	
None/below degree level	7 (38.9)
Degree level or higher	11 (61.1)
*Ethnicity*	
White British	18 (100.0)
*RUC2011 Rural–Urban Classification*	
Urban city and town	18 (100.0)
*Index of Multiple Deprivation Decile*	
Most deprived 10%	0 (0.0)
10%-20%	1 (5.6)
20%-30%	1 (5.6)
30%-40%	2 (11.1)
40%-50%	0 (0.0)
50%-60%	1 (5.6)
60%-70%	6 (33.3)
70%-80%	1 (5.6)
80%-90%	4 (22.2)
Least deprived 10%	2 (11.1)
*Cancer type*	
Breast	6 (33.3)
Urological	3 (16.7)
Skin	2 (11.1)
Lower gastrointestinal	4 (22.2)
Upper gastrointestinal	3 (16.7)

### Results in relation to recovery of subjective health and wellbeing framework

Various factors played a contributory role in the interruption of health and wellbeing and the subsequent steps to recovery of subjective health and wellbeing in the participants. These included problematic events, personal factors, healthcare factors, pre-existing factors and environmental factors. An explanation of the categories and sub-categories alongside example participant quotes and data can be found in Table 2.

**Table 2:** Explanation of subcategories within theoretical framework and example quotes

Factor	Subcategory	Explanation	Quote and/or example data
Problematic events	*Cancer diagnosis*	Being informed about the diagnosis of cancer and any challenges that arose due to it	*“I went to the GP who referred me to the clinic, I got an* *appointment through. So I went to the doctors on the 1st April, got my appointment through on the 14th April at the breast care. And they told me there and then that it was cancer.”* Female, 49
	*Cancer prognosis*	Explanation of the course cancer could take	*“So, you know he [consultant] assured me, he said reducing* *it, we would reduce the chances of getting cancer in that breast by fifty percent”* Female, 57
	*Cancer treatment*	Experience with primary cancer treatment and any challenges following	*“So, I sailed through* *radiotherapy. I had no* *problems at all with it. Other than tiredness and breathlessness I sailed through it.”* Female, 49
Pre-existing factors	*Age*	Age when interview was conducted and the influence it has had on their health	*“As I say, I’m 75… Most people can’t believe that I am the sort of age that I am and I’m still cutting around and doing things”* Male, 75
	*Gender*	Influence of gender on their cancer experience	*“Being a woman and going through what I did, to have the* *first lot of surgery and to sort of come out with half a body,* *or part of a body makes you feel just like, horrendous.”* Female, 54
	*Chronic conditions*	Any other long-term medical conditions as well as cancer	*“I’d had treatment for high blood pressure for a while, but* *apart from that I was pretty well.”* Male, 73
	*Employment*	Where participants currently/ previously worked and related experiences post cancer	*“I had three years on and off. I tried to go back [to work] after* *my chemotherapy but then I had to do the radiotherapy, so I* *was off again.”* Female, 49
	*Finances*	Financial situation prior to and post cancer diagnosis	*“I’m 76…had a good life, was earning six figures for years so* *I’ve got no money worries or anything like that.”* Male, 76
	*Deprivation*	As classified by the UK Office for National Statistics Index of Multiple Deprivation decile classification (1 = 10% most deprived to 10 = 10% least deprived.)	Least deprived 10% (Decile 10)
	*Relationship status*	Partner, married/divorced etc. and influence of cancer on relationship	*“I’m 76, married for 52 years to a wonderful wife and mother so what have I got to moan about.”* Male 76
	*Urban life*	Experience of living in an urban area, as classified by the ONS and reported on by the participants	*“We are not in the middle of the urban mass; but on the edge.”* Male, 65
Environmental factors	*Health provider usage*	Use of health services prior/post treatment, including compliance and follow-up care	*“Our doctor is just down the road; but if something is wrong we can call the duty manager. They have some basic health training.”* Male, 73
	*Social support*	Help from family, friends, and colleagues	*“The big positive was the support I had; the family coming close around.”* Male 68
	*Community support*	Help available from those in their local community, e.g. neighbours, local support groups	*“So in that sense, there would be much less support from the* *community, or you being known.”* Male 65
	*Travel*	Distance and methods used to travel to amenities	*“Here, there is a bus if I ever needed it.”* Female, 71
Personal factors	*Personality*	Personal characteristics or individual-level traits possessed by participants	*“I pretty much keep myself to myself, I talk to people if I see them, you know, out and about.”* Female, 44
	*General self-efficacy*	Participants confidence in being able to perform daily activities and self-manage their health and healthcare	*“I don’t think the fact that I go to Zumba is linked to the* *cancer as such, it’s just a more general looking after myself* *sort of thing.”* Female, 44
	*Responsibilities*	Factors that participants felt accountable towards, as well as protective factors	*“I’ve got three sons and five grandchildren.”* Female, 71
	*Mentality*	The attitude of participants	*“…it is nothing compared with* *if I hadn’t had radiotherapy. I wouldn’t have had any pain; but I wouldn’t be here.”* Male, 76
	*Resilience*	The ability to try to overcome hardship	*“I wasn’t prepared to sit back and just feel sorry for myself.”* Female, 54
	*Life events*	Events which may have had an influence on individual circumstances	*“My wife had breast cancer and they cured it”* Male, 71
Healthcare factors	*Cancer type*	The specific type of cancer diagnosed	*“Stage 3 cancer: a malignant melanoma”* Male, 68
	*Treatment type*	Type of primary cancer treatment undertaken	*“I had a full mastectomy on the* *right side. I then had chemotherapy, I then had the removal of the lymph nodes, I* *then had radiotherapy, and* *tamoxifen.*” Female, 49
	*Rapport with clinicians*	Nature of relationship between survivors and clinicians	*“Especially the radio* *[radiotherapy] people they were excellent like you know.”* Male, 76
	*Health literacy*	Understanding of health-related information	*“I don’t ask questions, I don’t know enough about it anyway, you know, I just do as I’m told and that’s it.”* Male, 75
	*Health resources*	Tools available/used by participants to manage their health and how well informed they felt during the process	*“And I knew I could ask anything as well.”* Female, 71

### Problematic events

A cancer diagnosis was an emotional, extremely challenging experience for most. Some participants were left fearing the worst and unable to truly comprehend the severity of the challenges associated with recovery. Some also felt that diagnostic delays were going to influence their recovery and reduce their chances of survival. The importance of keeping cancer patients informed of all details of their care was emphasised. Some participants felt that some of the healthcare staff they dealt with were blunt and impersonal. One survivor who had three separate cancer diagnoses describes how the quality and holistic approach to initial cancer care from diagnosis to treatment has progressed and improved since her first diagnosis: “And I can’t say how much difference and better that made me feel. And I’m thinking, the people that get it now, it is such a benefit to them, to experience that. If they had experienced when I had first got it, it was just awful, it was.” (Female, 54).

### Personal factors

Some remained resilient throughout the entire process, taking an attitude that they would not let cancer take control of their life, whilst others struggled mentally with adjusting to having had cancer and the ongoing effects of it, having trouble with confidence post-treatment and feeling as if they would never return to their pre-cancer selves. Life events, such as close friends and family going through cancer and their experiences, were also mentioned by participants. Many felt that having seen someone go through a similar experience aided their confidence in recovering. However, some participants had lost loved ones due to cancer and described the additional anxiety this caused them throughout their recovery process.

Most participants mentioned responsibilities and protective factors as reasons for them to persevere with their treatment and recovery. Older survivors with children or grandchildren described how “They are a great incentive to get better: you want to see them grow up.” (Male, 65). Participants who undertook volunteering, had other work or religious responsibilities mentioned the importance of these factors in encouraging them to take care of themselves to continue with these engagements.

### Healthcare factors

Cancer treatment and its influence on recovery varied based on the specific cancer stage and the treatment the participants underwent. Some felt that their treatment and cancer had little to no effect on them, however others highlighted the immense discomfort and trauma they experienced post-treatment. Rapport-building with clinicians formed a vital role in how survivors viewed their entire experience and, ultimately, how they dealt with any hurdles they may have encountered during their recovery. Those who felt that they had an overall worse experience with their care describe a more challenging journey to recover their subjective health and wellbeing. Emotionally, some participants felt they will never be able to truly recover following negative experiences.

Health literacy and resources impacted engagement in treatment and recovery post-treatment. Some cancer survivors had healthcare experience, and generally possessed a greater understanding of information they consumed throughout the course of their treatment. Paper leaflets and online resources were the most common health resources participants accessed. However, many participants “didn’t want to delve in too deep” (Male, 71) into some online resources, believing they would lead to more worrying, whilst others did not feel the need to do any research or were not computer literate enough to utilise these. Some attended support groups and they found them to be very useful and helped with dealing with their emotions. Others felt they did not need the additional support or did not know it existed, as they were not informed of it during healthcare visits. In addition, some participants describe a lack of resources available for their specific cancer when compared to other cancers.

### Pre-existing factors

Age-related problems with comorbidities, mobility, and cognition, can prove to be highly challenging in combination with a cancer diagnosis. Older participants expressed that they were grateful for living a good healthy life up until diagnosis and were ready to accept whatever outcome was meant for them. In contrast, others felt their age led to them being underestimated when trying to be independent by both healthcare professionals and their families. Those participants with mobility issues or previous psychological issues found it harder to try to get back to ‘normal’, struggling with self-management and physical activities.

Employment was shown to form part of a daily routine for those participants who worked and acted as a self-management strategy, helping achieve a sense of normality. Participants who were in work found they had to reduce their hours or sacrifice their jobs, but many returned to their original hours once they were well enough to do so. Throughout treatment, participants described receiving support from their employers, and conversely the self-employed participants reported finding the lack of financial or emotional support a traditional job confers challenging. Changes in the employment circumstances of participants following cancer treatment meant that some dealt with financial hardship on top of other problems. Some participants in a better financial position wished they had been better informed on private treatment options to expedite management.

Relationships were shown to be an essential source of support for participants, with partners acting as both a protective factor and an emotional outlet allowing the burden of a cancer diagnosis to be shared rather than being held solely by the cancer survivors. Sharing the burden mostly helped in supporting recovery, however a cancer diagnosis can also be challenging for close family and friends too in a psychosocial context, leaving them with uncertainty and worry around having to care for them constantly. For others, the challenges of cancer resulted in a breakdown of their relationship. Consequently, the recovery process was much more difficult with the added pressure of having to look after the household alone.

Urban residence was felt to confer some advantages in health management, whilst a minority felt it had no real influence on their health. Many participants commented on the convenience associated with having shops, services, parks, and social clubs near them and how it reduced mental and physical stress on the cancer survivors and their carers. Participants expressed the importance of their urban life on their health, with amenities close by and this also acted as a reason for living in these areas. Common problems participants mentioned regarding their urban residence were pollution and traffic, and a lack of green spaces, with some participants even considering moving to a more rural area due to the stress associated with such issues.

### Environmental factors

Environmental factors were found to have played a vital role in aiding self-management following primary cancer treatment and, consequently, recovery of subjective health and wellbeing. Some participants felt that they had “not much community cohesion” (Male, 65), whilst others appreciated how their urban residence “is a social area; to my mind a positive social thing… People meet for crafts and just talk.” (Male, 73), thus proving an avenue of social and mental relief during their recovery.

Participants emphasised the importance of the social support they received from family and friends, which was highly influential in allowing them to develop cancer-related self-efficacy. Participants found that by engaging with their social circles, they could keep busy and keep their minds off some of the mental aspects of cancer. Fear of recurrence and pain were described as prominent barriers to recovery. Survivors found support from others helped improve their confidence in dealing with such issues, although some participants also mentioned how they felt there was a sense of expectation of them returning ‘back to normal’ by family and friends, which resulted in increased mental burden.

Health provider usage also forms a recurring aspect of cancer survivors’ lives post-treatment, with many interviewees finding that they felt a sense of reassurance knowing that they had the self-efficacy to know whom to contact when concerns arose post-treatment. Some participants mention how once their treatment had ended, it “felt like stepping off a cliff” (Male, 65) and how “the worst thing with cancer is surviving it.” (Female, 49), suggesting further improvement was needed with the follow-up care to set up survivors for life beyond cancer.

Most urban residents mention how their location has made them better equipped to deal with their health, with more accessible travel to healthcare providers, “Being this close to the hospital has been a great reassurance. That has long-term psychological impact. Less stress.” (Male, 65). Better travel routes and public transport proved to be a significant benefit to most participants and made them more likely to engage with healthcare. As distances from treatment centres increased, travel became more of an issue when trying to access care, with some participants covering considerable distances, often being sent to multiple hospitals and cities for different stages of their treatment and having to rely on the generosity of friends and family to take them to appointments. Despite having a range of participants from both deprived and more affluent areas as measured via the Index of Multiple Deprivation, there were no overt or explicit differences in terms of experiences. Although there were more participants in this study resident in more affluent postcodes, when compared to the most deprived.

## Discussion

This study identified the challenges and opportunities that urban cancer survivors face following primary cancer treatment. The data analysed were incredibly rich and allowed for a wide range of lived experiences from those interviewed and has shown that aspects of urban life such as access to amenities, including healthcare facilities, travel, and social support aided their recovery. The pre-identified categories from the recovery of health and wellbeing framework [16] were successfully applied to the interview transcripts with urban cancer survivors. Therefore, this allowed for a greater understanding of how these factors interacted with the urban cancer survivors’ day-to-day lives post-treatment. These contributory factors from the adapted framework, all played varying roles in the recovery of health and wellbeing in these participants, highlighting that the recovery of subjective health and wellbeing was a journey unique to each individual.

Healthcare and environmental factors such as clinician rapport and travel helped participants to deal with some of the physical and psychosocial challenges of recovery (e.g., fear of recurrence and symptomatic pain relief). Often, participants’ urban residence meant close proximity to friends and family. This could be seen as a benefit of their urban living, with social support possibly acting as a protective factor and providing motivation for them to better engage with recovery. The results highlighted the main drawbacks of urban living including pollution and lack of green spaces. There were also mixed opinions on community cohesion within the urban areas; some participants found it very useful and prevalent, while others felt it was non-existent in their area.

The potentially negative effects of urban residence that can influence health and wellbeing [13, 36] were reinforced by this study, with participants highlighting the lack of accessible outdoor spaces and pollution as barriers to engaging in physical activity and exercise. Exercise forms part of self-management strategies in cancer survivors [37] and is shown to improve both mental and physical health [38], and therefore leading to better cancer-related self-efficacy; although, this can prove to be more challenging to engage with in some urban areas due to the lack of suitable outdoor spaces.

However, the qualitative lived experiences of the participants in this study suggest that participants also showed high levels of both general and cancer-related self-efficacy. This was heavily influenced by positive experiences with healthcare factors, such as their rapport with clinicians and environmental factors, including accessibility to amenities and social/community support. These findings are consistent with several studies which suggest a positive relationship between patient experiences with clinicians and social support and improvements in health [16, 39, 40].

Cancer support groups have been shown to result in a higher quality of life in cancer survivors [40], reducing the psychological symptoms of cancer survivorship and aiding in cancer-related self-management [41]. This was echoed by the findings of this study, with those that attended support groups finding them extremely useful.

Employment issues throughout the cancer experience can cause financial distress, especially in those who are self-employed, a population that may already be lacking in emotional and financial support as they make up such a small proportion of the UK workforce [42]. These experiences are reiterated across current literature, which suggests that self-employed cancer survivors across many countries experience comparatively worse quality of life and receive less support than their salaried counterparts [43–45]. Financial concerns have also been reported as a unique psychosocial need of rural cancer survivors [46] indicating that some psychosocial concerns can transcend urban or rural geography.

Whilst urban life helped aid self-management and recovery in this study, other contributory factors also played a crucial role in recovery. For example, rapport with clinicians and health services, resilience, and family caring responsibilities provided a strong foundation for participants to engage in self-management. Those with more positive experiences in these areas were shown to have felt that they were better managing their recovery. Further research into relationships between personal characteristics, healthcare rapport and cancer recovery may prove to be vital in understanding how these factors affect patient outcomes.

Despite its strengths, this study is limited in that it was self-selecting through postal questionnaires [21], and as such negative experiences may have been underrepresented. Although it could be argued the East Midlands region as a whole is somewhat representative of the UK in terms of urban/rural dynamics and ethnicity and deprivation [47, 48], in this study all participants were White British, it is thus important to note in this specific study the experiences only of this population are represented. Future cancer survivorship research needs to make concentrated efforts to recruit individuals from a range of different backgrounds. Despite reporting on some of the challenges that older participants could face there was no formal comparative analysis by age. It is likely that differences in life phases, particularly for those who are younger and are still balancing a career, as well as, caring for a young family will impact on recovery of health and wellbeing and future research should make concentrated efforts to explore this. Another limitation of this study is that the analysis and coding process was conducted primarily by one researcher, however it is argued that inter-rater reliability is not always needed to produce high-quality data, when reflexivity is employed [49, 50].

## Conclusion

This study is the first of its kind in applying Foster and Fenlon’s recovery of health and wellbeing framework [16] to an exclusively urban qualitative cancer survivorship dataset. A range of factors acted as facilitators for recovery in urban participants, including but not limited to; accessibility to healthcare and amenities, social support, and travel routes, all of which had an influence on the most critical aspects of recovery, such as cancer-related self-efficacy, coping and self-management strategies. Urban residence rarely acted as a barrier to recovery; however, concerns that more should be done to deal with pollution and its subsequent influence on health, as well as the lack of green spaces and community cohesion, were made evident in this study.

Ultimately, as well as urban living, a wide range of factors influenced recovery, especially rapport with clinicians and pre-existing and personal factors. It reinforces the fact that healthcare providers and policymakers need a more holistic approach to meet the complex needs of cancer survivors in urban settings post-cancer treatment. This could be achieved via outreach to cancer survivors and understanding the importance of continued follow-up care to set realistic expectations of the recovery of health and wellbeing.

## Data Availability

No datasets were generated or analysed during the current study.
